# Asclepias Incarnata in Gonorrhœa and Syphilis

**Published:** 1858-08

**Authors:** Wm. Hauser

**Affiliations:** Spier’s Turn-Out, Ga.


### ARTICLE III.

*Asclepias Incarnata in Gonorrhœa and Syphilis.* By WM. HAUSER, M. D., of Spier's Turn-Out, Ga.

Perhaps no country beneath the sun is so rich in valuable medicinal plants, as these same United States of ours; and in no part of these is the indigenous flora, so varied, beautiful, and useful, as in the Southern States. Here, at our very doors, from the Potomac to the Rio Grande, are found, in endless profusion, anodyne, cathartic, emetic, diuretic, diaphoretic, tonic, and alterative plants, &c., &c.; but a guilty apathy prevails among us about developing our own resources, and we prefer to import across long wastes of ocean, and from our enemies, at heavy cost, drugs far less valuable than those we might find at home, if we would only leave off wasting our time in hunting, fishing, &c., and engage with becoming industry and perseverence in the business of our profession. To do a little of my own duty in the premises, I offer this paper.

Six kinds of Asclepias are mentioned in Wood & Bache's Dispensatory, ed. of 1851, viz: Incarnata, Syriaca, Tuberosa, a Curassavica, Gigantea, and Vincetoxicum. Five of these appear to have been used, at different times, in the treatment of urinary and genital diseases. With the first only, I propose to deal in this article. And I will say, in passing, that I have never yet talked with a physician who professed to have any knowledge either of the physical or therapeutical properties of the plant in question. Every one could talk handsomely of successes in treating gonorrhœa, if not syphilis, in its different stages; and so, doubtless, they will continue to do, while Balsam Copaiba, Tr. Cubebs, Dulc Sp. Nitr., &c., maintain their potency; but cases constantly arise that are dreadfully perplexing, even to the best and most experienced physicians, and then it is that a groaning comes on for an enlargement of the domain of medical knowledge.

The physical properties of this plant being sufficiently described in the Dispensatory, no mention of them is necessary here. But very little—almost nothing—is said in that book, to which every young Doctor inevitably looks for information, about medical substances, concerning the therapeutic properties of it. I have found it, not only alterative, diaphoretic, and diuretic, but one of the finest aperients in the Materia Medica. Though this article was meant for a monograph, it is deemed best to state at this point, that I have used it with the happiest results in many forms of fever, but chiefly as an adjuvant to other medicines.

For the treatment of gonorrhœa and syphilis, I doubt if there is any thing equal to it now known to the medical fraternity. The root only is used, and the medical properties of that reside in the cortex. I have used, for many years, the tincture only, prepared in diluted alcohol, on account of the gum with which the root abounds.

℞ Asclep. Incar, ℥iv.

Diluted Alcohol, Oji.

After about 14 days' maceration, as with most drugs, it is prepared for use. I have generally prescribed it in a tablespoonfull: dose three times a day; i. e., before breakfast, dinner and supper. And this I have done, with very little regard to the stage of the disease, both in gonorrhœa and syphilis, however heterodox and unscientific it may appear to those nice critics in the medical fraternity who can theorize learnedly, but fail in practice.

CASES—GONORRHŒA.

*Case 1st.*—J. T., aet. 24, was afflicted with "*running of the reins*," (strange that all have this, and none of them venereal,) "*brought on by a strain;*" and it had afflicted him long and sorely, even to the extent of a change from white to greenish discharges. I put him on tr. of asclep. incarn. alone. But as his business required him to ride a great deal on horse-back, thus keeping up the irritation in the genital region, I think it was two weeks before I succeeded in curing him.

*Case 2d.*—R. F., aet. 28, of intemperate habits, as well as priaputic. He had the candor to say, laughing, "I dont care if you do know what ails me." A pretty wise conclusion in one calling on a Doctor to cure him of disease. A single bottle of ℥vjii, (if my memory be correct as to the size of the bottle) cured him.

*Case 3d.*—J. A. P., aet. 30, like Don Juan, had received a donation "from general subscription of the ladies." His business required daily riding on horse-back, but the Tr. Asclep. cured him, I think he said, in three days.

I deem it unnecessary to report any other cases of gonorrhœa successfully treated with this article, although they have accumulated satisfactorily in a series of years, *as civilization*, (as I tell the boys,) has advanced.

SYPHILIS.

B. N. had been looking the picture of the wo-begone and despairing for two or three months, seemingly afraid to call for help. Impelled by a feeling of pity, I at last urged him to call on some physician without delay. He placed himself under my treatment. I put him on my favorite Tr.—blistered over his mammary glands several times during the course, and had large quantities of mercurial ointment rubbed into them, after each removal of the cuticle. After two or three weeks' perseverance, I had the unspeakable happiness to see my patient restored to hope and to health. Despair fled from his soul, and that horrid, disgusting sallowness from his skin, and the bloom of the rose came back to his cheeks. This occurred in 1854, and my friend still lives and flourishes as though that "incurable" disease had never afflicted him.

Of the German plan of Syphilization, just now attracting attention, well may Dr. Jas. Bryan exclaim, "let any one imagine three hundred chancres produced for the cure of one!" If physicians will lay aside all indifference, and use the Asclepias Incarnata, especially in Gonorrhœa and Syphilis, I have no shadow of doubt that it will soon take rank in their estimation, with the very foremost remedies in our Materia Medica.

